# B-Mode Ultrasonography and Acoustic Radiation Force Impulse Elastography in Evaluation of Urothelial Carcinoma in Dogs

**DOI:** 10.3390/ani15091223

**Published:** 2025-04-26

**Authors:** Ana Paula Luiz De Oliveira, Bruna Bressianini Lima, Denise Jaques Ramos, Gabriela Castro Lopes Evangelista, Igor Cezar Kniphoff Da Cruz, Ricardo Andrés Ramirez Uscategui, Andrigo Barboza de Nardi, Marcus Antônio Rossi Feliciano

**Affiliations:** 1 Departamento de Clínica e Cirurgia Veterinária, Faculdade de Ciências Agrárias e Veterinárias, Universidade Estadual Paulista, Jaboticabal 14884-900, SP, Brazil; apl.oliveira@unesp.br (A.P.L.D.O.); bbressianinilima@gmail.com (B.B.L.); denise.97.jr@gmail.com (D.J.R.); andrigo.barboza@unesp.br (A.B.d.N.); 2 Departamento de Medicina Veterinária, Faculdade de Zootecnia e Engenharia de Alimentos, Universidade de São Paulo, Pirassununga 13635-900, SP, Brazil; gabriela.clopes@yahoo.com.br; 3 Departamento de Diagnóstico por Imagem, Universidade Federal de Santa Maria, Santa Maria 97105-900, RS, Brazil; igor.cruz@ufsm.br; 4 Departamento de Sanidad Animal, Facultad de Medicina Veterinaria y Zootecnia, Universidad del Tolima, Ibagué 730006299, Tolima, Colombia; ramirezuscategui@hotmail.com

**Keywords:** transitional cell carcinoma, urinary bladder, BRAF, ultrasound

## Abstract

Bladder tumors in dogs represent approximately 2% of all malignant lesions recorded, with urothelial carcinoma being the most common and aggressive form. This study aimed to characterize the elastographic properties of canine urothelial carcinoma using ARFI (Acoustic Radiation Force Impulse) elastography and to evaluate its diagnostic capacity. Seven dogs with urothelial carcinoma and nine healthy dogs were evaluated, comparing shear wave velocity (SWV) and bladder wall thickness between the groups. The results showed that SWV was significantly higher in dogs with urothelial carcinoma (median of 2.53 m/s) compared with healthy dogs (median of 1.41 m/s) (*p* = 0.0045). Bladder wall thickness was also greater in dogs with carcinoma (0.28 ± 0.05 cm) compared to healthy dogs (0.14 ± 0.26 cm) (*p* = 0.0003). Receiver operating characteristic (ROC) curve analysis revealed a high sensitivity (100%) and specificity (88.9%) for SWV in differentiating healthy and neoplastic tissues. These findings suggest that ARFI elastography is a promising tool for diagnosing urothelial carcinoma in dogs, offering a noninvasive and quantitative approach that can complement traditional diagnostic methods. ARFI elastography may aid in early diagnosis and clinical management, improving the prognosis of patients with bladder neoplasia.

## Simple Summary

Urothelial carcinoma is the most common and aggressive bladder tumor in dogs. Using ARFI (Acoustic Radiation Force Impulse) elastography, we found that shear wave velocity (SWV) and bladder wall thickness were significantly higher in dogs with urothelial carcinoma compared to healthy dogs. In addition, SWV showed a high sensitivity and specificity in differentiating healthy and neoplastic tissues. We suggest that ARFI elastography, as a noninvasive and quantitative approach, can be used as a promising tool for diagnosing urothelial carcinoma in dogs.

## 1. Introduction

Bladder tumors represent around 2% of neoplasms diagnosed in dogs, with urothelial carcinoma or transitional cell carcinoma being the most frequently identified [1,2,3]. Due to the invasive nature, metastatic potential, and frequent development in the bladder trigone, the prognosis of these neoplasms is unfavorable [4]. Histopathological examination can provide a definitive diagnosis and characterization of urothelial carcinoma; however, it is not routinely used, due to the discouragement of veterinarians from performing biopsies by the notable capacity of iatrogenic implantation during surgery or cystocentesis [2,5].

Since the relationship between neoplasm morphology and its clinical behavior is a key point in oncology, different imaging modalities have been widely used in veterinary practice to identify and monitor neoplasm evolution [2,6]. Two-dimensional (B-mode) ultrasonography is a highly accurate technique for detecting bladder lesions and, when performed systematically, may be superior to double-contrast cystography for identifying bladder masses [3]. It is a reliable tool for assessing the size of canine urinary bladder tumors [7] and can detect ultrasound lesions that can be used as prognostic indicators of urothelial carcinoma [8]. Despite being a noninvasive, accessible, and low-cost modality, the differentiation between benign and malignant lesions is still challenging [9].

Elastography is an advanced ultrasound technique that allows the evaluation of mechanical properties of tissues, particularly their stiffness, by measuring the shear deformation of tissues in one or both shear states after applying dynamic forces or gradual forces that are considered ’quasi-static’. The tissue deformation can be represented visually in an elasticity image (elastogram) or quantified locally in several ways [10]. Acoustic Radiation Force Impulse (ARFI) imaging is one way of observing the tissue displacement directly. Alternatively, the deformation itself can be calculated and displayed, resulting in what is known as strain elastography (SE). Finally, in dynamic protocols, the data can be used to record the propagation of shear waves, which can be used to calculate the regional values of their velocity (without generating images) using methods such as transient elastography (TE) and point shear wave elastography (pSWE), or to generate images of their velocity using methods as shear wave elastography (SWE), which includes 2D SWE and 3D SWE [10,11].

The use of elastography has now become widespread in veterinary medicine, particularly for the evaluation of various conditions of the urogenital system [12,13,14,15] and as an auxiliary method in the study of different neoplasms in canine species and with promising results in predicting lesion malignancy [16,17,18]. In humans, elastography is a useful tool in the evaluation of patients diagnosed with bladder masses, with malignant lesions being significantly stiffer than benign ones [19], as well as in the differentiation of low-grade and high-grade urothelial carcinoma [20].

To our knowledge, there are no studies using Acoustic Radiation Force Impulse (ARFI) elastography to evaluate urinary bladder disorders in dogs. Considering that the determination of tissue elastic properties allows a better evaluation of lesions, we hypothesized that ARFI elastography would provide reproducible quantitative data that would be able to aid in urothelial carcinoma identification, offering a noninvasive and accessible approach for veterinary clinical practice. Therefore, this study aimed to characterize the elastographic properties of urothelial carcinomas in dogs using ARFI elastography and verified its diagnostic potential by comparing healthy tissues and neoplastic urinary blader tissues stiffness.

## 2. Materials and Methods

### 2.1. Animals

This prospective, observational study conducted between September 2022 and March 2024 was approved by the Institutional Animal Care and Use Committee (protocol number 4280/22). In this study, two types of animals were enrolled, dogs with urothelial carcinoma (UTC group) and healthy dogs (CON group).

The UTC group included dogs treated by the oncology service of the “Governador Laudo Natel” University Veterinary Hospital and diagnosed with urothelial carcinoma by histopathological examination, using BRAF gene identification (PCR) in urine, or by cytological analysis, using urinary sediment or cytology brushing [1,21,22], who have not received any type of chemotherapy previously, after their owners provided signed consent for participation in this study. There were no restrictions regarding sex, age, or breed. The CON group included healthy dogs from the Nutrition and Nutritional Diseases Research Laboratory.

A detailed medical history was obtained for each patient, with particular attention given to any previous urinary tract disease. A clinical examination was performed and signs such as abdominal pain on palpation, stranguria, and urinary urgency were recorded. Finally, the dogs were referred to the radiology service for ultrasound examination.

### 2.2. Imaging Evaluation

Ultrasound examinations were performed using the ACUSON S2000^®^ equipment (Siemens, Munich, Germany), with a 9.0 MHz multifrequency linear transducer (9L4 Transducer, Siemens, Munich, Germany). Animals were previously depilated and positioned in dorsal or lateral recumbency according to the requirements of this study. All ultrasound examinations were performed by the same operator.

Initially, B-mode ultrasound was performed to assess the urinary bladder. The transducer was positioned in the caudal abdomen and after locating the organ, images were taken in longitudinal and transverse planes of its entire length. The bladder was assessed for its distension, thickness, regularity, integrity of the wall, general appearance, echogenicity, and presence or absence of masses or particles (urine) within or around it. Specifically in dogs of the UTC group, the evaluation of the bladder masses included dimensions (height and length), echotexture pattern (homogeneous or heterogeneous), location (apex, body, trigone, or diffuse), contours (regular or irregular), tumor shape (pedunculated or nonpedunculated), bladder wall involvement (involvement of the muscular layer of the bladder wall or loss of normal stratification), and echogenicity (hypoechoic, hyperechoic, or mixed) [8].

After B-mode examination, ARFI elastography was performed using Virtual Touch IQ software (Siemens, Munich, Germany). First, images of the bladder wall of all dogs and of the neoformations in dogs of the UTC group were obtained. The quality of the images was tested using a software indicator in which homogeneous and greenish images indicated high quality, while heterogeneous and yellowish images indicated low quality; when the latter occurred, the imaging was repeated until it reached a standard quality. Based on this image quality, a qualitative and quantitative evaluation was carried out. In the qualitative one, the deformability (deformable or non-deformable) and the relative stiffness of the tissues (in the color elastogram, blue regions indicate fewer rigid structures, green regions indicate intermediate stiffness, and red regions indicate more rigid structures) were classified. In the quantitative evaluation, at least five square regions of interest (ROIs of 1 × 1 mm) were randomly positioned in each evaluated tissue in order to include the maximum area, and the software immediately provided the values of the shear wave velocity (SWV) of each of these ROIs [23,24]. In the CON group, the same evaluation sequence was applied, making SWV measurements in the dorsal region of the bladder wall.

### 2.3. Statistical Analysis

Statistical analysis was performed using GraphPad Prism software (version 8.0.0 for Windows, GraphPad Software, Boston, MA, USA). Initially, tissue shear wave velocities (SWV) of different regions of interest (ROIs) of the bladder wall and masses were compared to each other using the Kruskal–Wallis’s test. If this analysis did not show significance, the median SWV of the ROIs were calculated and used for subsequent analyses. Bladder SWVs and wall thickness were compared between the UTC group and CON group using the Mann–Whitney test. If this test was significant, the SWV and/or wall thickness were subjected to a discriminative power analysis to identify the presence of neoplasia through receiver operating characteristic (ROC) curve analysis, and the cut-off value, sensitivity, specificity, likelihood ratio, and area under the curve (AUC) were calculated for this diagnosis, using the logistic regression model by means of the Wilson/Brown method. Qualitative variables were analyzed subjectively (relative stiffness) and compared by Fisher’s exact test (presence or absence). Statistical significance was considered when *p*-value < 0.05, and data are presented as the median ± interquartile range (IQR).

## 3. Results

Nine healthy dogs for the CON group and seven dogs with urothelial carcinoma for the UTC group were included in this study. In the UTC group, four dogs had the diagnosis confirmed by identification of the BRAF gene in urine, one by histopathological examination, and two by mixed methods (cytological analysis of urinary sediment, BRAF, and histopathological).

The UTC group was composed of five females (5/7; 71%) and two males (2/7; 29%), with a mean age of 12 ± 3 years, and belonging to various races: Basset Hound (2/7), Border Collie (1/7), German Shepherd (1/7), Yorkshire Terrier (1/7), and mongrel (2/7). The CON group was composed of six females (6/9; 67%) and three males (3/9; 33%), with a mean age of 5 ± 2 years, all of the beagle breed.

All dogs of the UTC group showed clinical signs of dysuria and abdominal pain, as well as urinary incontinence or dribbling urination, while in the CON group, no dogs showed clinical signs. Furthermore, urine color varied between orange and red in 6/7 patients of the UTC group, and it was yellow in all animals in the CON group.

**Figure 1:**
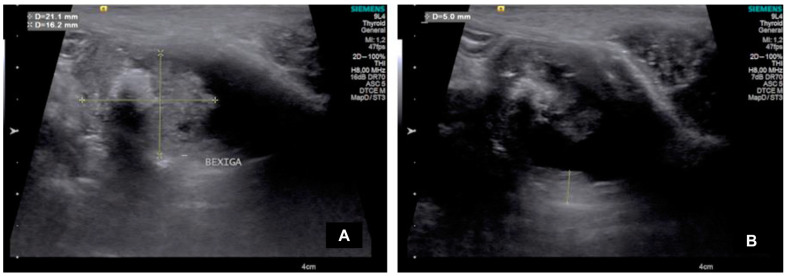
B-mode ultrasound images of the urinary bladder in patients with urothelial carcinoma. (**A**) B-mode ultrasound evaluation of pedunculated bladder formation with invasion of the bladder wall and dimensions (height and length). (**B**) Evaluation and measurement of bladder wall thickness in a canine patient with urothelial carcinoma.

In the CON group, the bladder was full of urine, with a normal appearance and regular wall, while in the UTC group, 3/7 dogs had an irregular bladder wall, and all animals had an abnormal appearance. In both groups, the bladder content was predominantly anechoic and homogeneous (Figure 1).

All neoformations had a pedunculated form with muscular wall invasion, and its echogenicity was hyperechogenic in 5/7 dogs and mixed in 2/7 with heterogeneous characteristics and irregular borders. The mass size, echotexture, and location varied among animals (Table 1); however, its average size was 1.72 ± 1.04 cm in height and 3.08 ± 1.01 cm in length.

**Figure 2:**
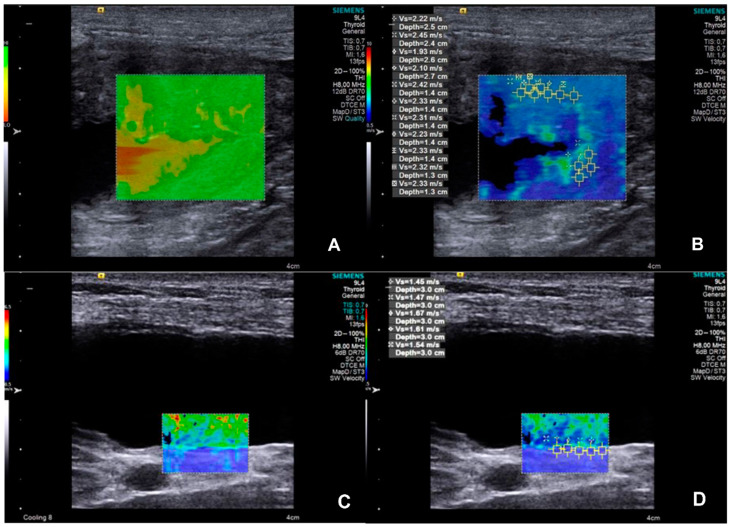
Images of ARFI (Acoustic Radiation Force Impulse) elastography of bladder neoformation in a dog diagnosed with urothelial carcinoma (**A**,**B**) and in a healthy dog (**C**,**D**). (**A**) A quality map image for ARFI evaluation of bladder neoformation; homogeneous and greenish areas indicate high quality of the exam, while heterogeneous and yellowish/reddish areas indicate low quality. (**B**) An elastogram for qualitative (colors scale) and quantitative evaluation (tissue stiffness shown as Shear Wave Velocity in m/s). Calipers of regions of interest (ROIs) were performed randomly. (**C**) A quality map image for ARFI evaluation of the dorsal bladder wall; homogeneous and greenish areas indicate high quality of the exam, while heterogeneous and yellowish/reddish areas indicate low quality. (**D**) An elastogram for qualitative (color scale) and quantitative evaluation (tissue stiffness shown as Shear Wave Velocity in m/s). Calipers of regions of interest (ROIs) were performed randomly.

**Figure 3:**
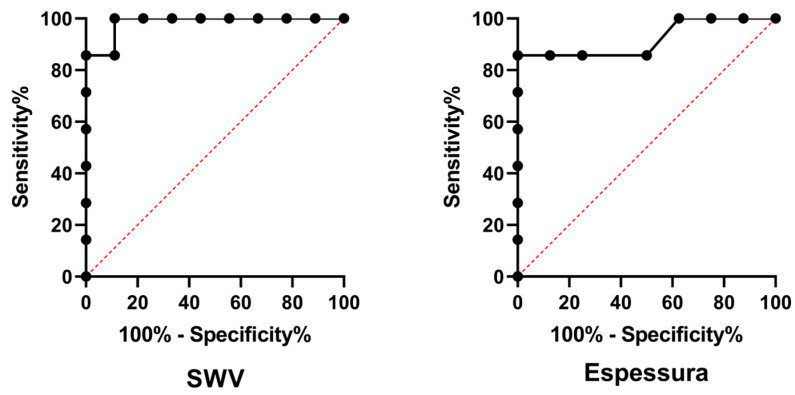
Receiver operating characteristic (ROC) curves comparing the diagnostic sensitivity and specificity of shear wave velocity (SWV) on ARFI elastography and bladder wall thickness for identify urothelial carcinoma in dogs.

The SWV measurements of different ROIs (Figure 2) resulted in similar values between each (*p* = 0.2925); consequently, the median SWV of the bladder wall was used for the posterior analysis. The SWV and thickness of the bladder wall resulted in higher values (*p* = 0.0045 and 0.0003, respectively) in the UTC patients, indicating that both bladder wall SWV and thickness showed discriminative power for identifying canine urothelial carcinoma (*p* = 0.0012 and 0.0065, respectively). Diagnostic features and median values are presented in Table 2 and illustrated in Figure 3.

## 4. Discussion

This study provides a preliminary characterization of the elastographic properties of a healthy bladder and urothelial carcinomas in dogs using the ARFI elastography technique as a noninvasive and accessible approach for veterinary clinical practice. The analysis of the SWV, with a median of 2.53 m/s in the group of dogs with urothelial carcinoma compared with 1.41 m/s in the healthy dogs, and of bladder wall thickness, with 0.28 ± 0.05 cm in the UTC group, in contrast to 0.14 ± 0.26 cm in the CON group, revealed that both variables have promising potential to detect bladder neoplasms and that ARFI elastography may be an important screening tool for urothelial carcinoma. The establishment of reference values based on the characterization of healthy dog tissue is a fundamental step. The cut-off value of 1.585 m/s for SWV identified in this study may provide a starting point for future differentiation between benign and malignant lesions.

These results corroborate previous research in neoplasms from other organs, where greater tissue stiffness has been associated with histopathological changes, such as increased cell density, disordered proliferation, and stromal disorganization [16,25]. Elastography is a feasible technique for objectively and subjectively characterizing the liver, spleen, and kidneys in cats [26] and dogs [27]. It was a feasible technique for assessing the liver and spleen stiffness of healthy dogs [28] and may be useful for predicting the presence of hepatic fibrosis in dogs with hepatic disease [29]. Studies performed in humans with liver and kidney diseases have shown that ARFI elastography has high sensitivity for detecting increased tissue stiffness, which is correlated with malignancy [30,31]. The thickening is consistent with tumor infiltration seen in invasive carcinomas, as reported in previous studies that used ultrasonography and computed tomography for the evaluation of bladder neoplasms [1]. Thus, this tumor infiltration, reflected by the thickening of the bladder wall, is indicative of malignant behavior, highlighting the importance of ultrasound evaluation as a complementary tool for both the diagnosis and staging of urothelial carcinoma.

The analysis of the ROC curves in this study revealed a sensitivity of 100% for SWV and 85.7% for bladder wall thickness, indicating that the technique has potential for excellent diagnostic capacity. The high sensitivity observed for SWV is consistent with the results of studies that used ARFI elastography for the diagnosis of breast and prostate cancers in humans, where the technique was demonstrated to be a valuable tool for the early detection of malignant lesions [11,32,33].

In addition to the quantitative findings, the clinical signs reinforce the fact that urothelial carcinomas are diagnosed late and that the clinical signs resemble those observed in cystitis, making early diagnosis difficult. The animals in the diseased group presented a variety of clinical signs, such as pain on palpation, urine with altered color, and difficulty in keeping the bladder full, which are frequently associated with bladder neoplasms [2]. Incontinence is commonly observed in patients with urothelial carcinoma [34]; one hypothesis is that it occurs precisely due to the increase in rigidity with a consequent decrease in compliance [35]. These clinical signs, combined with the observation of heterogeneous bladder masses with irregular borders, corroborate previous studies indicating that these characteristics are associated with a more reserved prognosis [8]. Furthermore, the reduced dependence on operator subjectivity compared with conventional ultrasound makes ARFI elastography a quantitative and reproducible tool, which is essential for the standardization of diagnoses [17,24].

Additionally, the fact that ARFI elastography can detect subtle tissue changes even before the lesions become macroscopically visible represents a significant advance in the diagnosis of several pathologies [36]. Several studies have evaluated the application of ARFI elastography in the differentiation of malignant and benign tumors in different tissues of dogs, including splenic tumors, cutaneous neoplasms, and the identification of metastatic lymph nodes in bitches with mammary neoplasia, demonstrating that the technique can be effective in identifying lesions with malignant characteristics, contributing to a more accurate diagnosis and clinical decision making [18,24,37,38]. The results of the present study, associated with the historical applicability of ARFI elastography in the differentiation between healthy and diseased tissues, demonstrate the application of the technique in the evaluation of canine bladder neoplasms, where high tissue stiffness is associated with the presence of urothelial carcinoma.

Future studies evaluating bladder wall stiffness in cases of neoplasia and comparing it with bladder wall stiffness in cases of chronic cystitis, where the wall is chronically thickened, as well as in cases of benign formations, such as polyps, are important in defining whether there is a difference between neoplasia and benign lesions, between neoplasia and normal tissue, and between benign lesions and normal tissue and to establish the cut-off values for each of them. Finally, a study carried out in children with cystitis, where they compared the values of the stiffness of healthy and diseased tissue by two-dimensional shear wave elastography, also identified a correlation between the SWV and wall thickness [39].

ARFI elastography and B-mode ultrasound examination can provide information that, although not strictly diagnostic, is essential for the early diagnosis process, providing information that can be used as a guide for decision making by both the veterinary oncologist and the guardian/owner. Therefore, it is important to explore in future research the combination of ARFI elastography with other diagnostic techniques, such as the detection of genetic mutations, such as the BRAF mutation. The association between molecular methods and imaging diagnostics could further improve diagnostic accuracy and allow a more personalized approach in the treatment of patients with urothelial carcinoma [40]. The literature suggests that the integration of techniques can significantly increase the sensitivity and specificity of diagnoses, especially in aggressive and difficult-to-detect neoplasms [18].

Although the results of this study are promising, it is important to recognize its limitations. The limited number of cases evaluated, especially with few histopathological results, is one of the main limitations, which may affect the generalization of the results. In addition, it was not possible to evaluate the local invasion (urethra and prostate) and metastatic behavior of the tumor in the population studied, as well as the classification and staging of the tumors or serial evaluations that would allow the monitoring and evolution of these patients. Future studies are encouraged and should include larger and more diverse samples, both in terms of the number of animals and racial diversity, to validate the findings and ensure that the ARFI technique is effective in a wide range of patients [21].

## 5. Conclusions

This study demonstrated that ARFI elastography is a tool in the diagnosis of urothelial carcinoma in dogs, providing quantitative information that can complement traditional approaches. It suggests that new studies in the area should be carried out to reinforce the findings and expand the clinical use of ARFI elastography in veterinary practice. New studies based on this one should be carried out to expand its application for differentiating neoplastic and non-neoplastic diseases.

## Figures and Tables

**Table 1:** Ultrasound aspects of masses found in the urinary bladder of seven dogs diagnosed with urothelial carcinoma.

Dog	Height (cm)	Length (cm)	Echotexture	Echogenicity	Location
1	0.88	3.53	Heterogeneous	Mixed	Dorsal
2	2.76	4.49	Heterogeneous	Hyperechogenic	Ventral, trigone e prostatic urethra
3	1.62	2.11	Heterogeneous	Hyperechogenic	Cranial (ventrodorsally)
4	3.75	3.63	Heterogeneous	Mixed	Cranial (ventrodorsally)
5	0.60	1.41	Homogeneous	Hyperechogenic	Trigone
6	1.24	2.51	Heterogeneous	Hyperechogenic	Dorsal
7	1.19	3.87	Heterogeneous	Hyperechogenic	Ventral

**Table 2:** Ultrasound and ARFI (Acoustic Radiation Force Impulse) elastography quantitative characteristics, bladder wall thickness, and Shear Wave Velocity (SWV) in healthy dogs (CON group = 9) and in dogs with urothelial carcinoma (UTC group = 7). Diagnostic performance variables of these variables are also presented to identify urothelial carcinoma.

Variable	Group	Median ± IQR	*p*-Value	Cut-Off Value
SWV of the bladder wall (m/s)	CON	1.41 ± 0.50	0.0045	>1.585
	UTC	2.53 ± 2.11		
	Sensitivity (%)	Specificity (%)	Likelihood ratio	AUC
	100.0	88.89	9.000	0.984
Bladder wall thickness (cm)	Group	Median ± IQR	*p*-value	Cut-off value
	CON	0.14 ± 0.26	0.0003	>0.165
	UTC	0.28 ± 0.05		
	Sensitivity (%)	Specificity (%)	Likelihood ratio	AUC
	85.71	87.50	6.857	0.919

AUC: area under the curve.

## Data Availability

The raw data supporting the conclusions of this article will be made available by the authors on request.

## References

[1] Knapp D.W., McMillan S.K. (2013). Tumors of the urinary systems. Withrow & MacEwen’s Small Animal Clinical Oncology.

[2] Fulkerson C.M., Knapp D.W. (2015). Management of transitional cell carcinoma of the urinary bladder in dogs: A review. Vet. J..

[3] Mutsaers A.J., Widmer W.R., Knapp D.W. (2003). Canine Transitional Cell Carcinoma. J. Vet. Intern. Med..

[4] Knapp D.W., Ramos-Vara J.A., Moore G.E., Dhawan D., Bonney P.L., Young K.E. (2014). Urinary Bladder Cancer in Dogs, a Naturally Occurring Model for Cancer Biology and Drug Development. ILAR J..

[5] Brambilla E., Govoni V.M., Cavalca A.M.B., Laufer-Amorim R., Fonseca-Alves C.E., Grieco V. (2022). Grading Systems for Canine Urothelial Carcinoma of the Bladder: A Comparative Overview. Animals.

[6] Macrì F., Di Pietro S., Mangano C., Pugliese M., Mazzullo G., Iannelli N.M., Angileri V., Morabito S., De Majo M. (2018). Quantitative evaluation of canine urinary bladder transitional cell carcinoma using contrast-enhanced ultrasonography. BMC Vet. Res..

[7] Honkisz S.I., Naughton J.F., Weng H.Y., Fourez L.M., Knapp D.W. (2018). Evaluation of two-dimensional ultrasonography and computed tomography in the mapping and measuring of canine urinary bladder tumors. Vet. J..

[8] Hanazono K., Fukumoto S., Endo Y., Ueno H., Kadosawa T., Uchide T. (2014). Ultrasonographic findings related to prognosis in canine transitional cell carcinoma. Vet. Radiol. Ultrasound.

[9] Léveillé R., Biller D.S., Partington B.P., Miyabayashi T. (1992). Sonographic investigation of transitional cell carcinoma of the urinary bladder in small animals. Vet. Radiol. Ultrasound.

[10] Bamber J., Cosgrove D., Dietrich C.F., Fromageau J., Bojunga J., Calliada F., Cantisani V., Correas J.-M., D’Onofrio M., Drakonaki E.E. (2013). EFSUMB guidelines and recommendations on the clinical use of ultrasound elastography. Part 1: Basic principles and technology. Ultraschall Med..

[11] Cui X.W., Li K.N., Yi A.J., Wang B., Wei Q., Wu G.G., Dietrich C.F. (2022). Ultrasound elastography. Endosc. Ultrasound.

[12] Caspanello T., Zappone V., Orlandi R., Sforna M., Boiti C., Sinagra L., Donato G., De Majo M., Iannelli N.M., Troisi A. (2025). Shear Wave Elastography Evaluation of Testicular Stiffness in Dogs Affected by Testicular Pathology. Animals.

[13] Zappone V., Iannelli N.M., Sinagra L., Donato G., Quartuccio M., Cristarella S., De Majo M., Caspanello T. (2024). Assessment of testicular stiffness in fertile dogs with shear wave elastography techniques: A pilot study. Front. Vet. Sci..

[14] Feliciano M.A.R., Maronezi M.C., Simões A.P.R., Maciel G.S., Pavan L., Gasser B., Silva P., Uscategui R.R., Carvalho C.F., Canola J.C. (2016). Acoustic radiation force impulse (ARFI) elastography of testicular disorders in dogs: Preliminary results. Arq. Bras. Med. Vet. Zootec..

[15] Feliciano M.A.R., Maronezi M.C., Simões A.P., Uscategui R.R., Maciel G.S., Carvalho C.F., Canola J.C., Vicente W.R. (2015). Acoustic radiation force impulse elastography of prostate and testes of healthy dogs: Preliminary results. J. Small Anim. Pract..

[16] Park H., Park J.Y., Ahn S.H., Chon C.Y., Han K.H., Kim S.U. (2013). Characterization of focal liver masses using acoustic radiation force impulse elastography. World J. Gastroenterol..

[17] Feliciano M.A.R., Uscategui R.A.R., Maronezi M.C., Simões A.P.R., Silva P., Gasser B., Pavan L., Carvalho C.F., Canola J.C., Vicente W.R.R. (2017). Ultrasonography methods for predicting malignancy in canine mammary tumors. PLoS ONE.

[18] Da Cruz I.C.K., Carneiro R.K., De Nardi A.B., Uscategui R.A.R., Bortoluzzi E.M., Feliciano M.A.R. (2022). Malignancy prediction of cutaneous and subcutaneous neoplasms in canines using B-mode ultrasonography, Doppler, and ARFI elastography. BMC Vet. Res..

[19] Dede O., Teke M., Daggulli M., Penbegül N. (2018). Use of Acoustic Radiation Force Impulse Elastography to Discrimination Benign and Malignant Masses for Bladder. Int. J. Radiol. Imaging Technol..

[20] Huang X.Z., Zhou A.Y., Liu M.W., Zhang Y., Xu P. (2021). Shear Wave Elasticity Differentiation Between Low-and High-Grade Bladder Urothelial Carcinoma and Correlation with Collagen Fiber Content. J. Ultrasound Med..

[21] Childress M.O., Adams L.G., Ramos-Vara J.A., Freeman L.J., He S., Constable P.D., Knapp D.W. (2011). Results of biopsy via transurethral cystoscopy and cystotomy for diagnosis of transitional cell carcinoma of the urinary bladder and urethra in dogs: 92 cases (2003-2008). J. Am. Vet. Med. Assoc..

[22] Yamasaki H., Uematsu Y., Okano K., Ichikawa M., Tei M., Hirabayashi M., Hirao H. (2022). Establishment and characterization of urothelial carcinoma cell lines with and without BRAF mutation (V595E) in dogs. Vitr. Cell. Dev. Biol.-Anim..

[23] Abreu T.G.M., Feliciano M.A.R., Renzo R., Kobashigawa K.K., Chacaltana F.D.Y.C., Crivelaro R.M., Silveira C.P.B., Cruz N.R.N., Aldrovani M., Maronezi M.C. (2018). Acoustic radiation force impulse elastography of the eyes of brachycephalic dogs. Arq. Bras. Med. Vet. Zootec..

[24] Maronezi M.C., Carneiro R.K., Da Cruz I.C.K., De Oliveira A.P.L., De Nardi A.B., Pavan L., Feliciano M.A.R. (2022). Accuracy of B-mode ultrasound and ARFI elastography in predicting malignancy of canine splenic lesions. Sci. Rep..

[25] Nightingale K., McAleavey S., Trahey G. (2003). Shear-wave generation using acoustic radiation force: In vivo and ex vivo results. Ultrasound Med. Biol..

[26] White J., Gay J., Farnsworth R., Mickas M., Kim K., Mattoon J. (2014). Ultrasound elastography of the liver, spleen, and kidneys in clinically normal cats. Vet. Radiol. Ultrasound.

[27] Jeon S., Lee G., Lee S.K., Kim H., Yu D., Choi J. (2015). Ultrasonographic elastography of the liver, spleen, kidneys, and prostate in clinically normal beagle dogs. Vet. Radiol. Ultrasound.

[28] Tamura M., Ohta H., Nisa K., Osuga T., Sasaki N., Morishita K., Takiguchi M. (2019). Evaluation of liver and spleen stiffness of healthy dogs by use of two-dimensional shear wave elastography. Am. J. Vet. Res..

[29] Tamura M., Ohta H., Shimbo G., Osuga T., Sasaki N., Morishita K., Kagawa Y., Takiguchi M. (2019). Usefulness of noninvasive shear wave elastography for the assessment of hepatic fibrosis in dogs with hepatic disease. J. Vet. Intern. Med..

[30] Gallotti A., D’Onofrio M., Romanini L., Cantisani V., Mucelli R.P. (2012). Acoustic Radiation Force Impulse (ARFI) ultrasound imaging of solid focal liver lesions. Eur. J. Radiol..

[31] Yoǧurtçuoǧlu B., Damar Ç. (2021). Renal elastography measurements in children with acute glomerulonephritis. Ultrasonography.

[32] Kumm T.R., Szabunio M.M. (2010). Elastography for the characterization of breast lesions: Initial clinical experience. Cancer Control.

[33] Meng W., Zhang G., Wu C., Wu G., Song Y., Lu Z. (2011). Preliminary results of acoustic radiation force impulse (ARFI) ultrasound imaging of breast lesions. Ultrasound Med. Biol..

[34] Reed L.T., Knapp D.W., Miller M.A. (2013). Cutaneous metastasis of transitional cell carcinoma in 12 dogs. Vet. Pathol..

[35] Kendall A., Byron J.K., Westropp J.L., Coates J.R., Vaden S., Adin C., Oetelaar G., Bartges J.W., Foster J.D., Adams L.G. (2024). ACVIM consensus statement on diagnosis and management of urinary incontinence in dogs. J. Vet. Intern. Med..

[36] Furuichi Y., Moriyasu F., Taira J., Sugimoto K., Sano T., Ichimura S., Miyata Y., Imai Y. (2013). Noninvasive diagnostic method for idiopathic portal hypertension based on measurements of liver and spleen stiffness by ARFI elastography. J. Gastroenterol..

[37] Feliciano M.A.R., Maronezi M.C., Crivellenti L.Z., Crivellenti S.B., Simões A.P.R., Brito M.B.S., Garcia P.H.S., Vicente W.R.R. (2015). Acoustic radiation force impulse (ARFI) elastography of the spleen in healthy adult cats–a preliminary study. J. Small Anim. Pract..

[38] Silva P., Uscategui R.A.R., Maronezi M.C., Gasser B., Pavan L., Gatto I.R.H., Feliciano M.A.R. (2018). Ultrasonography for lymph nodes metastasis identification in bitches with mammary neoplasms. Sci. Rep..

[39] Durmaz M.S., Yorulmaz A., Durmaz F.G., Arslan S. (2021). Utility of 2-Dimensional Shear Wave Elastography for Assessment of the Bladder Wall in Children with Acute Cystitis. J. Ultrasound Med..

[40] Mochizuki H., Shapiro S.G., Breen M. (2015). Detection of BRAF mutation in urine DNA as a molecular diagnostic for canine urothelial and prostatic carcinoma. PLoS ONE.

